# In Vitro Effects of Papaverine on Cell Migration and Vascular Endothelial Growth Factor in Cancer Cell Lines

**DOI:** 10.3390/ijms23094654

**Published:** 2022-04-22

**Authors:** Daniella Anthea Gomes, Anna Margaretha Joubert, Michelle Helen Visagie

**Affiliations:** Department of Physiology, School of Medicine, Faculty of Health Sciences, University of Pretoria, Private Bag X323, Gezina, Pretoria 0031, South Africa; daniella.a.d.gomes@gmail.com (D.A.G.); annie.joubert@up.ac.za (A.M.J.)

**Keywords:** papaverine, cell migration, cancer, pFAK, VEGF B, VEGF R1, VEGF R2

## Abstract

Papaverine (PPV) is a benzylisoquinoline alkaloid isolated from *Papaver somniferum* that exerts antiproliferative activity. However, several questions remain regarding the biochemical pathways affected by PPV in tumourigenic cells. In this study, the influence of PPV on cell migration (light microscopy), expression of vascular endothelial growth factor (VEGF) B, VEGF R1, VEGF R2, and phosphorylated focal adhesion kinase (pFAK) were investigated using spectrophotometry in MDA-MB-231-, A549- and DU145 cell lines. The migration assay revealed that, after 48 h, PPV (100 µM) reduced cell migration to 81%, 91%, and 71% in MDA-MB-231-, A549-, and DU145 cells, respectively. VEGF B expression was reduced to 0.79-, 0.71-, and 0.73-fold after 48 h of exposure to PPV in MDA-MB-231-, A549- and DU145 cells, while PPV exposure of 48 h increased VEGF R1 expression in MDA-MB-231- and DU145 cells to 1.38 and 1.46. A fold decrease in VEGF R1 expression was observed in A549 cells to 0.90 after exposure to 150 µM. No statistically significant effects were observed on VEGF R2- and FAK expression after exposure to PPV. This study contributes to the understanding of the effects of a phytomedicinal alkaloid compound in cancer cells and may provide novel approaches to the application of non-addictive alkaloids.

## 1. Introduction

The use of herbal- and traditional medicines dates back as far as 5000 years ago across numerous civilisations with ancient texts indicating the use of at least 800 plants, with the earliest texts the instructing use of herbal remedies found in China, dating back to 2800 BC. Roughly 121 pharmaceutical products in use today have been formulated based on traditional knowledge developed over the last century [1]. Plants and plant-derived compounds have attracted significant interest and attention and have become more incorporated into modern medicines [1]. Global focus on phytomedicines that strengthen and repair biological systems without toxic side effects has developed as a separate industry, with approximately 80% of the global population relying on plant-derived medicines for primary health care due to limited side effects [1]. Examples of compounds in current use include morphine which is used as a painkiller and quinine which is used to overcome fevers and artemisinin in the treatment of malaria [1]. Papaverine (PPV) is another example of a phytomedicinal compound that is currently used clinically as a vasodilator in the treatment of cerebral vasospasms [2,3,4,5,6,7]. 

Benzylisoquinoline alkaloids (BIAs) are a group of metabolites isolated from plant materials and are currently being investigated for possible medicinal properties for use in the health care sector [8]. PPV is one of the BIAs obtained from *Papaver somniferum*. However, morphine and codeine are the most common BIAs isolated from the same plant [7]. Research exploring the cytotoxic effects of PPV on tumourigenic cell lines, including breast ductal-carcinoma (T47D)-, colorectal carcinoma (HT 29)-, fibrosarcoma (HT1080), triple negative breast carcinoma (MDA-MB-231), adenocarcinoma alveolar cancer (A549), and prostate carcinoma (DU145) has shown that PPV selectively and dose-dependently reduced the cell viability of tumourigenic cell lines with no significant effects observed in non-tumourigenic cell lines namely embryonic fibroblasts (NIH 3T3) T-cells and the non-tumourigenic human fibroblast (NHF) cell line [6,7,9,10,11,12,13,14]. Previous research shows that PPV induced a reduction in cell viability in a time- and dose-dependent manner in several tumourigenic cell lines including MDA-MB-231-, A549-, and DU145 cells, accompanied by aberrant changes in the production of hydrogen peroxide (H_2_O_2_), a reactive oxygen species (ROS) mostly produced in the mitochondria [12]. Although these studies have shown that PPV exerts antiproliferative activity in tumourigenic cells, the mechanisms of action exerted by PPV are still not fully understood [15,16].

**Figure 1:**
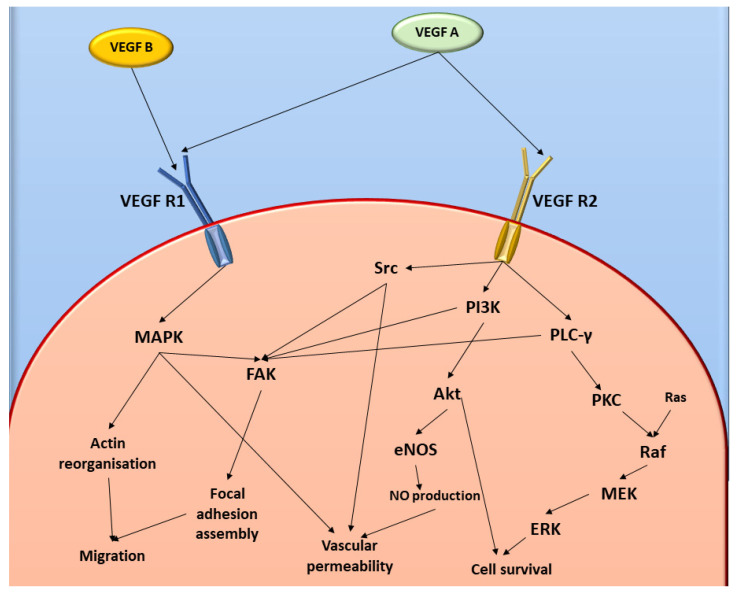
Diagrammatic representation of VEGF and FAK cell signalling pathways. Image was designed by DA Gomes using Microsoft^®^ Office PowerPoint (Microsoft Office enterprise 2007, 2006 Microsoft Corporation, Redmond, WA, USA).

Some studies have suggested that PPV is a phosphodiesterase 10A (PDE10A) inhibitor and results in the accumulation of adenosine 3′,5′-cyclic monophosphate (cAMP) which accounts for the vasodilatory effects that PPV exerts [15,17,18,19]. The effects of cAMP on tumour progression, mitochondrial functions, and the transcription of oncogenes, including p53, are extensive, with cAMP accumulation resulting in increased activity of pathways including the phosphatidylinositol-3-kinase/ protein kinase B (PI3K/Akt) pathway [18,19,20,21,22,23,24,25,26,27]. The PI3K/Akt pathway is a pro-survival pathway that can regulate multiple hallmarks of cancer, including continuous cell proliferation, metastasis, and tumour angiogenesis (Figure 1) [18,19,20,21,22,23,24,25,26,27]. Upregulation of this pathway can ultimately result in increased vascular endothelial growth factor (VEGF) ligand and receptor expression [28]. The VEGF receptor pathway is crucial in tumour angiogenesis and consists of 5 related ligands, namely placental growth factor (PlGF), VEGF A, VEGF B, VEGF C, and VEGF D which differentially bind to three receptor tyrosine kinases (VEGF R1, VEGF R2, and VEGF R3) [11,29,30,31]. Hypoxia-related angiogenesis typically involves ligands VEGF A and VEGF B and receptors VEGF R1 and VEGF R2 [29,30,31]. However, research indicates that VEGF B functions as a survival factor for multiple different types of vascular cells, including endothelial cells and pericytes, which aids in the stabilising of the vasculature. Furthermore, studies indicate negligible involvement of VEGF B in neovasculature formation [29].

In prostate cancer, the presence of VEGF ligand expression is frequently increased and is regulated by various stimulants, including tumour necrosis factors (TNF) α- and β and interleukin-1 (IL-1) [32]. IL-1 is a cytokine that is upregulated in response to inflammation and is associated with upregulated VEGF expression. Furthermore, TNF, an inflammatory cytokine, is a strong mediator of VEGF expression, the presence of both TNF and IL-1 in the tumour microenvironment may play a role in tumour vascularisation [32]. Studies have demonstrated that the expression of VEGF ligands in breast cancer cells is upregulated compared to non-tumourigenic cells. Furthermore, VEGF R2 expression is upregulated more in metastatic breast cancer compared to non-metastatic breast cancer, even though the increased expression of VEGF R2 is observed in both tumourigenic cell types. In addition, VEGF R2 is overexpressed more prominently in breast cancer compared to VEGF R1 expression [33]. Studies exploring the expression of VEGF in lung cancer have yielded contradicting results, with studies indicating varying levels of expression, including low, moderate, or high, with high expressivity typically associated with a worse prognosis [34]. 

**Figure 2:**
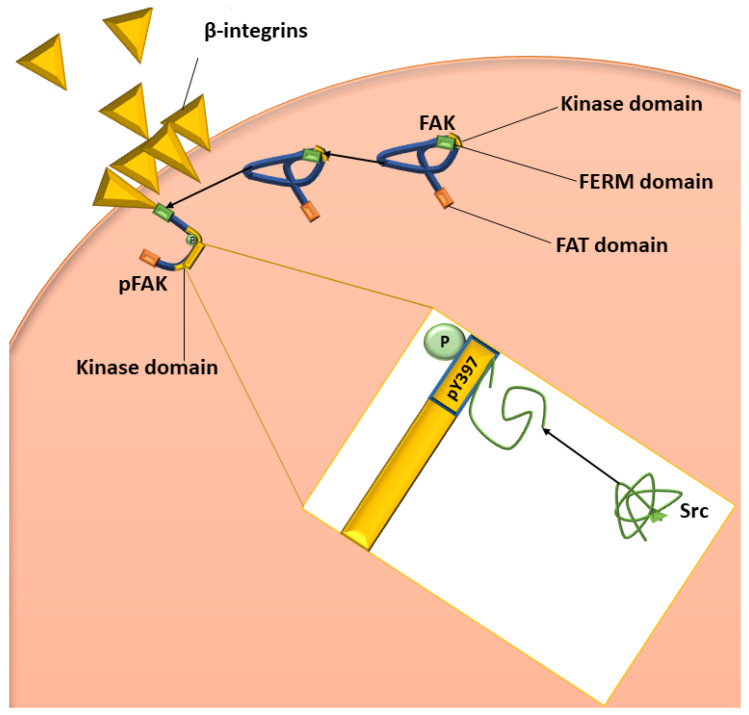
Diagrammatic representation of the activation of FAK and Src. The green bar on the blue FAK molecule represents the FERM domain, the orange bar represents the FAT domain, and the yellow bar represents the kinase domain. β-integrin clustering initiates FAK to bind to the cytoplasmic tail of the integrin, resulting in the unfolding of FAK, allowing autophosphorylation of pY397 (shown in the yellow box). Once FAK has been phosphorylated and therefore activated, Src can bind to pY397, which results in the unfolding of Src and, therefore, the activation of Src. Image was designed by DA Gomes using Microsoft^®^ Office PowerPoint (Microsoft Office enterprise 2007, 2006 Microsoft Corporation, Redmond, WA, USA).

Vascular permeability is a significant contributor to the tumour microenvironment, with overexpression of VEGF ligands and receptors resulting in hyperpermeable vasculature leading to an increase in hypoxic regions within tumourigenic tissues, which further stimulates VEGF expression [11,29,30,31]. Vascular permeability is also mediated by focal adhesion tyrosine kinase (FAK), a protein-tyrosine kinase, which is activated by VEGF and integrins through increased phosphorylation of the activation loop within the FAK protein structure located in focal contacts (Figure 2) [35,36,37]. FAK is essential in the signalling network between growth factors, including VEGF and other adhesion role-players such as β-integrins, with the downstream effects of these networks influencing vascular permeability, cell proliferation, and migration [35,37]. The autophosphorylation of FAK, facilitated by the clustering of β-integrins at pY397, is a crucial autophosphorylation site of FAK activation since the kinase domain is only capable of activating FAK once autophosphorylation has occurred [31,33,38,39]. In the event of cells forming attachments to adjacent cells, integrins will cluster and facilitate the binding of the erythrocyte band 4.1, ezrin, radixin, moesin homology (FERM) domain to cytoplasmic tails of β-integrin. Then, the FERM domain will release the kinase domain leading to the unfolding of the FAK protein to expose the kinase domain, thus allowing the autophosphorylation of pY397 and the subsequent activation of FAK (Figure 2) [31,39]. It has been suggested that there is a significant difference in the expression of FAK between non-tumourigenic and tumourigenic cells, with most studies indicating increased expression of FAK messenger ribonucleic acid (mRNA) in several cancer types, including invasive breast cancer, colon cancer, and prostate cancer, implicating that FAK may be involved in the progression of tumours [33,38,39,40]. Furthermore, overexpression of FAK is seen more within highly metastatic and invasive tumourigenic cell types [41]. FAK is also associated with cell migration since it functions as a motility regulator that possibly influences downstream pathways, including rat sarcoma (RAS) expression, and thus, FAK upregulation is associated with the promotion of cell migration [41]. 

The inhibition or alterations to FAK signalling and expression may affect cell survival by disrupting the attachment of cells to the membrane of the adjacent cell since FAK signalling is involved in cellular adhesions [40,41]. Moreover, due to the alterations to vascular permeability during tumour progression, typically due to the upregulation of angiogenesis, FAK is consequently localised to areas of vascular hyperpermeability [31]. Thus, VEGF-related activation of FAK results in increased recruitment of FAK which subsequently promotes cell migration [42]. However, the signal transduction and specific molecular components involved in the FAK pathway are yet to be confirmed, along with the influence of VEGF in mediating hypoxia-induced FAK activation remaining elusive and requires further investigation [31,42]. To date, the effects of PPV on VEGF ligands and receptors and FAK is yet to be reported and thus require further investigation [3,6].

It has been reported that PPV inhibits tumourigenic cell growth; however, the effects on biochemical pathways involving VEGF signalling remain unclear. Therefore, the current study is one of the first studies to investigate the influence of PPV on cell migration. Phosphorylated FAK (pFAK) and VEGF expression was investigated in a triple negative breast cancer cell line (MDA-MB-231), adenocarcinoma alveolar cancer cell line (A549), and a prostate cancer cell line (DU145). This will aid the understanding of the biochemical pathways that PPV effects and will improve the understanding of the use of PPV on tumourigenic cell lines. Understanding the effect of PPV on biological pathways such as VEGF and cell migration will improve cancer researchers’ understanding of PPV as a phytomedicinal compound and contribute to the existing knowledge regarding the influence of a naturally occurring compound in tumourigenic cells. Understanding the phytomedicinal compounds and determining the benefits of these compounds in comparison to current synthetic treatments may develop novel treatment options with reduced side effects and potentially improve survival. This may complement existing therapies and be beneficial or improve future therapeutic options. 

## 2. Results

### 2.1. Cell Migration Using Scratch Assay (Light Microscopy)

The effects of PPV on cell migration were investigated after 18, 24, and 48 h using the scratch assay, where the change in the width of each scratch over time was assessed and quantified since cells may migrate into the exposed surface of the scratch. The relative percentage migration compared to cells at 0 h exposure was determined for each treatment at 18, 24, and 48 h.

**Figure 3:**
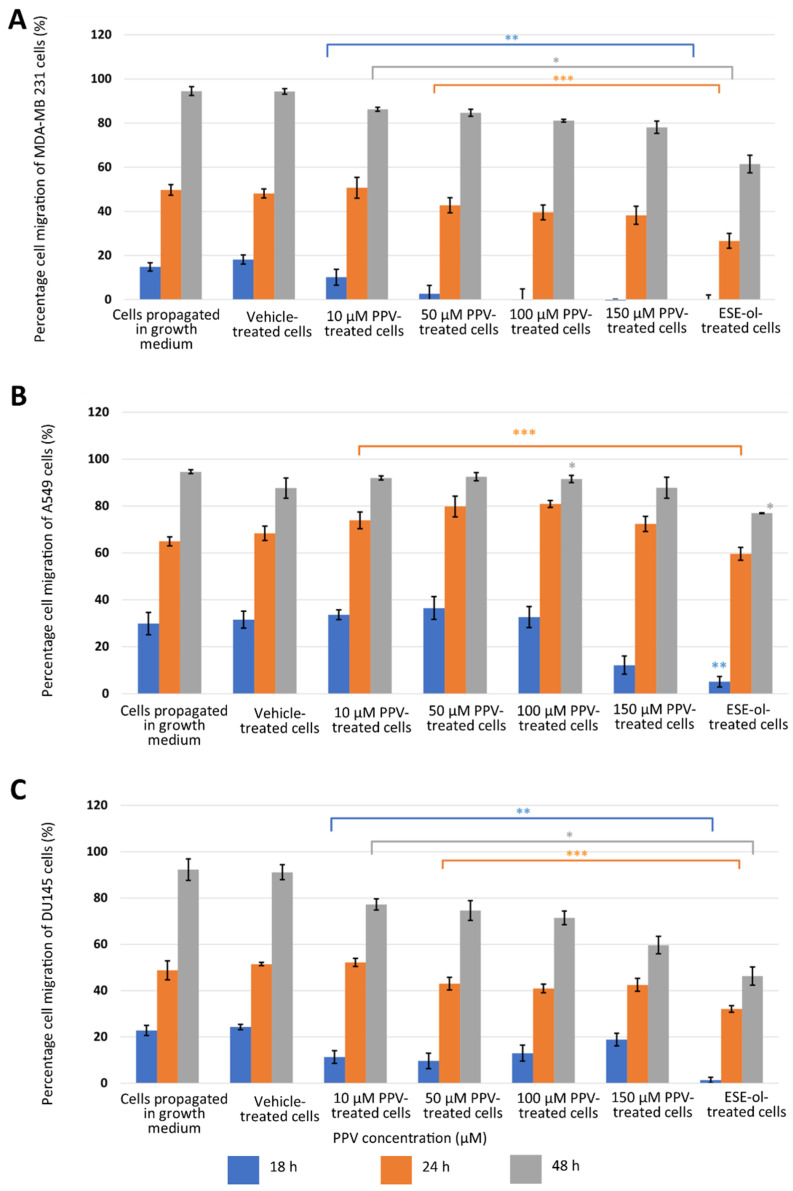
The relative percentage migration compared to MDA-MB-231 (**A**), A549 (**B**), and DU145 (**C**) cells at 0 h exposure when cells were exposed to PPV (10–150 µM). The average of three independent experiments is represented by the graph with error bars indicating standard deviation. The blue bar represents the percentage of the cell migration of the cells after 18 h of exposure, the orange bar represents the percentage of cell migration of cells after 24 h of exposure, and the grey bar represents the percentage of cell migration of cells after 48 h of exposure. The statistical significance of cell migration after 18 h of exposure is indicated with an asterisk (**), the statistical significance of cell migration after 24 h of exposure is indicated with two asterisks (***), and the statistical significance of cell migration after 48 h of exposure indicated with three asterisks (*). The positive control for the migration assay-included cells exposed to 2-ethyl-17-hydroxy-13-methyl-7,8,9,11,12,13,14,15,16,17-decahydro-6-cyclopenta[a]phenanthren-3-yl sulphamate (ESE-ol) (0.4 µM) for 48 h. Statistical significance is represented by an * when using the student *t*-test with a *p* value of 0.05 compared to cells propagated in complete growth medium.

Results indicated that PPV inhibited migration in all three cell lines in a time- and dose-dependent manner. MDA-MB-231 cells propagated in complete growth medium migrated 15% over 18 h and 50% over 24 h (Figure 3A). However, cells exposed to 10, 50, 100, and 150 µM of PPV migrated 10%, 3%, 0%, and 0% over 18 h and 50%, 43%, 40%, and 38% over 24 h. Furthermore, MDA-MB-231 cells propagated in complete growth medium migrated 94% over 48 h. However, cells exposed to 10, 50, 100, and 150 µM of PPV migrated 86%, 85%, 81%, and 78% over 48 h. The results obtained in the migration assay indicate that exposure to PPV inhibits the migration of MDA-MB-231 cells significantly in a dose-dependent manner over time, where exposure to increasing concentrations of PPV correlates with a decrease in percentage migration relative to 0 h. A549 cells propagated in a complete growth medium migrated 30% over 18 h and 64% over 24 h (Figure 3B). Cells exposed to 10, 50, 100, and 150 µM of PPV migrated 34%, 36%, 33%, and 12% over 18 h and 74%, 80%, 81%, and 72% over 24 h. Furthermore, A549 cells propagated in complete growth medium migrated 95% over 48 h. However, cells exposed to 10, 50, 100, and 150 µM of PPV migrated 92%, 92%, 91%, and 88% over 48 h. DU145 cells propagated in complete growth medium migrated 23% over 18 h and 49% over 24 h (Figure 3C). However, cells exposed to 10, 50, 100, and 150 µM of PPV migrated 11%, 10%, 13%, and 19% over 18 h and 52%, 43%, 41%, and 42% over 24 h. DU145 cells propagated in complete growth medium migrated 90% over 48 h. However, cells exposed to 10, 50, 100, and 150 µM of PPV migrated 77%, 75%, 71%, and 59% over 48 h. The results obtained in the migration assay indicate that exposure to PPV inhibits the migration of DU145 cells significantly in a dose-dependent manner over time where exposure to increasing concentrations of PPV correlates with a decrease in percentage migration relative to 0 h. In addition, the inhibitory effects of PPV on the percentage of cell migration was more prominent in MDA-MB-231 and DU145 cells in comparison to A549 cells. Therefore, these results indicate that an increase in PPV concentration correlates with a decrease in cellular migration and is observed in MDA-MB-231 and DU145 cells and maintained after exposure to PPV at all time points.

### 2.2. Detection of Vascular Endothelial Growth Factor Using ELISA (Spectrophotometry)

**Figure 4:**
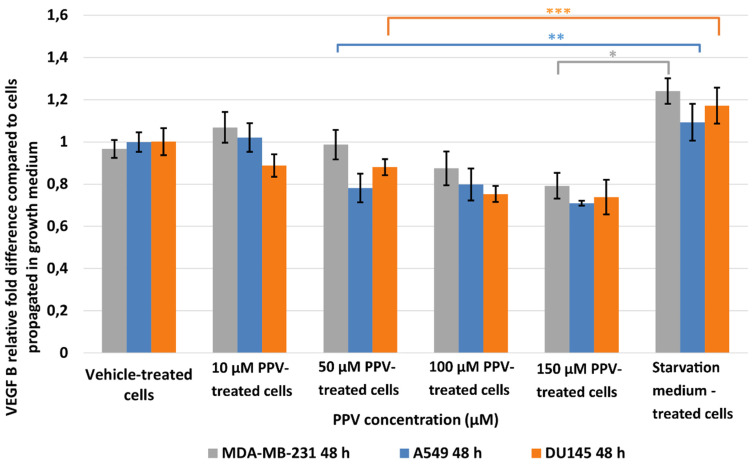
Spectrophotometry results of VEGF B ELISA demonstrating the effects of PPV (10–300 µM) on proliferation on MDA-MB-231 cells compared to A549- and DU145 cell lines at 48 h. The average of three independent experiments is represented by the graph with error bars indicating standard deviation. The statistical significance of MDA-MB-231 cells is indicated with an asterisk (*), the statistical significance of A549 cells is indicated with two asterisks (**), and the statistical significance of DU145 cells is indicated with three asterisks (***). Statistical significance is represented by an * when using the student *t*-test with a *p* value of 0.05 compared to cells propagated in complete growth medium.

The effects of PPV on VEGF expression were investigated in MDA-MB-231-, A549-, and DU145 cells. Results indicated that a statistically significant fold decrease to 0.79 was observed in VEFG B expression in MDA-MB-231 cells exposed to 150 µM PPV for 48 h compared to cells propagated in a complete growth medium (Figure 4). Furthermore, exposure to 50, 100, and 150 µM of PPV in A549 cells for 48 h resulted in a statistically significant fold decrease to 0.78, 0.79, and 0.71 when compared to cells propagated in a complete growth medium. Exposure to PPV for 48 h demonstrated a statistically significant fold decrease to 0.88, 0.75, and 0.73 when exposed to 50, 100, and 150 µM PPV in DU145 cells compared to cells propagated in a complete growth medium. The results, therefore, indicate that exposure to increasing concentration of PPV correlates with a decrease in VEGF B expression after 48 h in all three cell lines. Furthermore, results indicate that the PPV affects VEGF B expression in a cell line-specific manner, with the A549- and DU145 cell lines most prominently affected by PPV.

**Figure 5:**
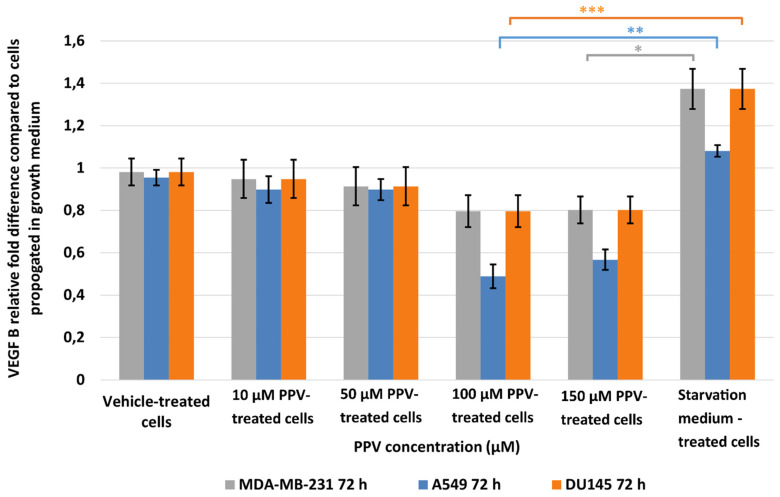
Spectrophotometry results of VEGF B ELISA demonstrating the effects of PPV (10–300 µM) on proliferation on MDA-MB-231 cells compared to A549- and DU145 cell lines at 72 h. The average of three independent experiments is represented by the graph with error bars indicating standard deviation. The statistical significance of MDA-MB-231 cells is indicated with an asterisk (*), the statistical significance of A549 cells is indicated with two asterisks (**), and the statistical significance of DU145 cells is indicated with three asterisks (***). Statistical significance is represented by an * when using the student *t*-test with a *p* value of 0.05 compared to cells propagated in complete growth medium.

Results indicated that a statistically significant fold decrease to 0.80 in VEGF B expression was observed in MDA-MB-231 cells after exposure to 150 µM for 72 h when compared to cells propagated in a complete growth medium (Figure 5). Furthermore, exposure to PPV for 72 h in A549 cells resulted in a statistically significant fold decrease to 0.49 and 0.57 after exposure to 100 and 150 µM PPV compared to cells propagated in a complete growth medium. Exposure to 100 and 150 µM PPV for 72 h demonstrated a statistically significant fold decrease to 0.79 and 0.88 in DU145 cells compared to cells propagated in a complete growth medium. Similar to the data obtained after 48 h exposure, these results indicate that exposure to increasing concentrations of PPV correlates with a decrease in the expression of VEGF B after 72 h. These results also indicate that the change in VEGF B expression is cell line-specific, with the A549 cell line most affected by PPV. Since 48 h exposure to PPV yielded a significant decrease in VEGF B expression similar to the results yielded after 72 h exposure to PPV, subsequence experiments investigating VEGF R1, VEGF R2, and FAK expression included cells exposed to PPV (10–150 µM) for 48 h only.

**Figure 6:**
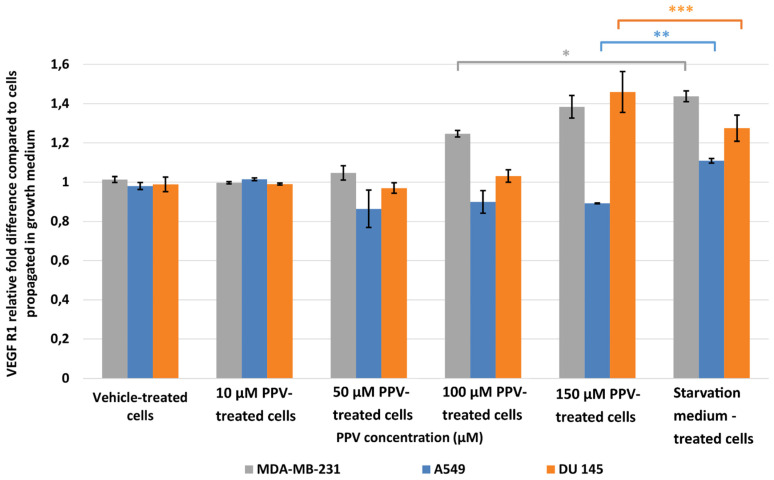
Spectrophotometry results of VEGF R1 ELISA demonstrating the effects of PPV (10–300 µM) on proliferation on MDA-MB-231 cells compared to A549- and DU145 cell lines at 48 h. The average of three independent experiments is represented by the graph with error bars indicating standard deviation. The statistical significance of MDA-MB-231 cells is indicated with an asterisk (*), the statistical significance of A549 cells is indicated with two asterisks (**), and the statistical significance of DU145 cells is indicated with three asterisks (***). Statistical significance is represented by an * when using the student *t*-test with a *P* value of 0.05 compared to cells propagated in complete growth medium.

Results indicated that a statistically significant fold increase to 1.25 and 1.38 in VEGF R1 expression was observed in MDA-MB-231 cells after exposure to 100 and 150 µM PPV for 48 h compared to cells propagated in a complete growth medium (Figure 6). Furthermore, exposure to 150 µM PPV for 48 h resulted in a statistically significant fold decrease to 0.90 in A549 cells compared to cells propagated in a complete growth medium. Exposure to 150 µM PPV for 48 h led to a statistically significant fold increase to 1.46 in DU145 cells compared to cells propagated in a complete growth medium. These results indicate that the change in VEGF R1 expression is cell-line specific, with MDA-MB-231- and DU145 cell lines most affected by higher concentrations of PPV.

**Figure 7:**
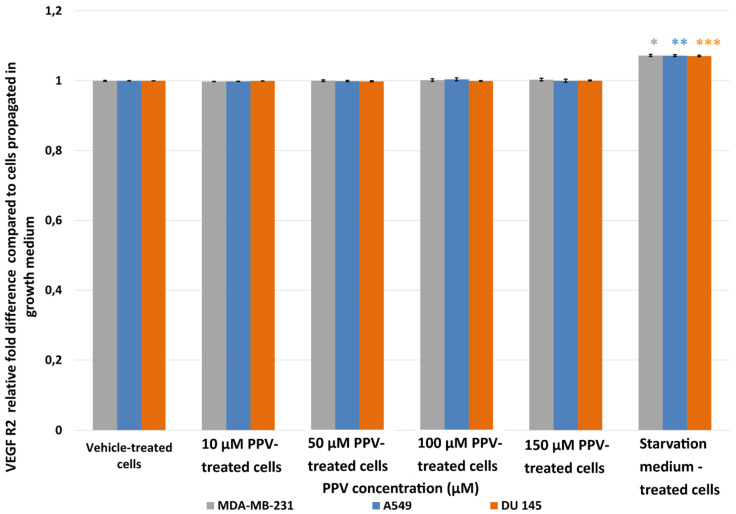
Spectrophotometry results of VEGF R2 ELISA demonstrating the effects of PPV (10–300 µM) on proliferation on MDA-MB-231 cells compared to A549- and DU145 cell lines at 48 h. The average of three independent experiments is represented by the graph with error bars indicating standard deviation. The statistical significance of MDA-MB-231 cells is indicated with an asterisk (*), the statistical significance of A549 cells is indicated with two asterisks (**), and the statistical significance of DU145 cells is indicated with three asterisks (***). Statistical significance is represented by an * when using the student *t*-test with a *p* value of 0.05 compared to cells propagated in complete growth medium.

Results showed that no statistically significant changes in VEGF R2 were induced after exposure to PPV for 48 h in any cell line. As a statistically significant increase in VEGF R2 was observed in the positive control, starvation medium-treated cells where cells were propagated in a 50% complete growth medium and 50% PBS, it is possible to suggest that PPV does not cause alterations to VEGF R2 when compared to cells propagated in complete growth medium (Figure 7). 

### 2.3. Determination of FAK (Phospho) [pY397] Using ELISA (Spectrophotometry)

The influence of PPV on phospho (pY397) FAK (pFAK) expression was evaluated by using a FAK- (Phospho) [pY397] specific ELISA kit and spectrophotometry [35,43]. Autophosphorylation of FAK occurs only when Y397 is exposed, and this typically occurs when cells form attachments and the FAK protein unfolds to change its conformation to expose the kinase domain. When Y397 is autophosphorylated to pY397, FAK is activated. Therefore, the expression of pFAK measures the expression of active FAK within the cell [35,43]. 

**Figure 8:**
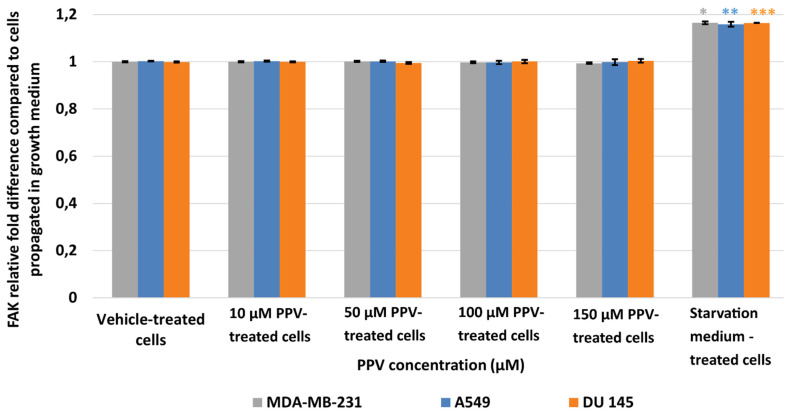
Spectrophotometry results of FAK ELISA demonstrating the effects of PPV (10–300 µM) on proliferation on MDA-MB-231 cells compared to A549- and DU145 cell lines at 48 h. The average of there independent experiments is represented by the graph with error bars indicating standard deviation. The statistical significance of MDA-MB-231 cells is indicated with an asterisk (*), the statistical significance of A549 cells is indicated with two asterisks (**), and the statistical significance of DU145 cells is indicated with three asterisks (***). Statistical significance is represented by an * when using the student *t*-test with a *p* value of 0.05 compared to cells propagated in complete growth medium.

Results showed that no statistically significant changes were induced as a result of 48 h exposure to PPV (Figure 8). In addition, a statistically significant increase in pFAK was observed in the positive control, starvation medium-treated cells where cells were propagated in a 50% complete growth medium and 50% PBS. Thus, data from the current study suggests that PPV exposure does not significantly affect pFAK expression at any concentration after 48 h exposure. 

## 3. Discussion

A recent study indicated that PPV exerts antiproliferative activity accompanied by oxidative stress and cell cycle abnormalities in a time- and dose-dependent manner in several tumourigenic cell lines [14]. However, the effects exerted by PPV, a vasodilator, on cellular migration, VEGF ligands and receptors, and pFAK expression remain unclear. Consequently, the effects PPV exerts on cellular migration and VEGF ligands and receptors, as well as pFAK, were investigated in the current study [15]. Results indicate that although PPV does not completely inhibit migration, there is a dose-dependent effect (10–100 µM) on migration where PPV does reduce cell migration in all three cell lines. Furthermore, results indicated that effects are cell line- and dose-dependent, with the most prominent effects observed in the A549 cell line in comparison to the MDA-MB-231- and DU145 cell lines. A decrease was observed in cell migration in the MDA-MB-231- and DU145 cells after 48 h exposure to PPV while an increase in cell migration was observed in the A549 cells after 48 h exposure to PPV. This indicates that PPV possibly promotes cell migration in A549 cells meanwhile reducing cell migration in MDA-MB-231- and DU145 cells, further suggesting the effects of PPV on cell migration are cell line dependent. This study is the first to report the effects of PPV on cell migration in MDA-MB-231-, A549, and DU145 cells.

The current study also explored the effects of PPV on pFAK and indicated that PPV does not significantly affect pFAK expression. FAK is a significant contributing signalling molecule to migration and cellular motility and functions as a motility regulator. Therefore, the upregulation of FAK is often associated with the upregulation of cell migration [44]. However, studies investigating the functions of FAK and FAK autophosphorylation have shown that even when FAK autophosphorylation is inhibited, and subsequently FAK activation is inhibited, tumourigenic cells are still capable of migration [35,38]. This suggests that although FAK autophosphorylation and FAK activation are involved in cell migration, there are alternative pathways that can be utilised by cells to migrate. It has been suggested that these mechanisms are controlled and activated by integrins. However, this alternative mechanism is not entirely understood and requires further research [35,38]. As the present study found that PPV does not exert any effects on the expression of autophosphorylated FAK but did indicate that alterations to cellular migration occurred, it is possible that cell migration is influenced by PPV through another pathway and not the FAK signalling cascade [39,40,44]. However, further investigation is required in future studies to unravel how PPV inhibits cell migration.

In addition, the present study also indicated that PPV exhibits dose- and cell line-dependent effects on VEGF B and VEGF R1 expression in all three cell lines. Exposure to PPV in MDA-MB-231 cells resulted in a decrease in VEGF B expression and an increase in VEGF R1 expression. Additionally, exposure to PPV in A549 cells resulted in a decrease in VEGF B and VEGF R1 expression. Exposure to PPV in DU145 resulted in a decrease in VEGF B and an increase VEGF R1. VEGF B is one of five VEGF ligands and has been implicated in hypoxia-related angiogenesis and the promotion of vascular cell survival. Hypoxia is a reduction or the impairment of oxygen tension in tissues and is a common abnormality observed in tumour progression [41,42]. Increased hypoxia induces the upregulation of VEGF B during tumour progression, and hypoxic levels typically stimulate the overexpression of VEGF B [45]. Consequently, it has been suggested that this overexpression results in the formation of neovasculature that are hyperpermeable and erratic in their structure and architecture [45,46]. VEGF B plays a significant role in both the sprouting of vasculature and the promotion of vascular survival, ensuring that the new vessels formed within the tumour survive [29,45,47,48]. Furthermore, VEGF B has been linked to pro-survival signalling in multiple different cell types indicating that the function of VEGF B is cell type or tissue type-specific [29,47]. 

However, the exact function of VEGF B is still a topic of debate with some studies suggesting it is a survival molecule while others suggest it is an angiogenic factor [28,29,32,34,47,48,49,50,51]. One possible mechanism through which VEGF B functions is through interacting with VEGF R1 [48]. Studies have shown that VEGF B has a higher binding affinity to VEGF R1 than other VEGF receptors, with the binding of VEGF B to VEGF R1 facilitating specific signalling depending on cell type [29,47,48,50]. VEGF R1 has been shown to affect homeostasis and vascular development with the overexpression of VEGF R1 inhibiting VEGF R2 and the phosphorylation of extracellular signal-related kinase (ERK) [48]. The binding of VEGF B to VEGF R1 leads to the activation of the intracellular Akt signalling pathway, which activates the nuclear factor κ-light-chain-enhancer (NFκB) [52]. Previous studies investigating the effects of PPV on tumourigenic cell lines have implicated that PPV downregulates the expression of PI3K, phosphorylated Akt, and NFκB in a dose-dependent manner in prostate carcinoma (PC-3) cells [9]. However, the mechanisms that result in the downregulation of PI3K, phosphorylated Akt, and NFκB were not identified. In the present study, a decrease in the expression of VEGF B and VEGF R1 was observed in A549 cells, whilst a decrease in the expression of VEGF B and an increase in the expression of VEGF R1 were observed in MDA-MB-231- and DU145 cells, as the binding of VEGF B to VEGF R1 activates the PI3K/Akt and the NFκB, it is possible that the downregulation of these pathways may be as a result of VEGF B downregulation [9]. However, further investigation into the connection between these pathways and the effects of PPV on the downregulation of these pathways need to be conducted. 

The present study found no statistically significant effects on VEGF R2 expression. VEGF R2 has been shown to mediate growth and survival signalling through the PI3K/Akt pathway and has been implicated in the activation of several downstream proteins, such as FAK. However, previous research has shown that PPV downregulates PI3K/Akt pathway [9]. Therefore, the effects of PPV may not be mediated through the VEGF R2 pathway, which may be bypassed as there is no change in VEGF R2 expression. VEGF R2 and FAK signalling are linked, with FAK requiring VEGF R2 signalling to activate expression. The present study found that the expression levels of both FAK and VEGF R2 were unaffected by PPV, further supporting the link between VEGF R2 and FAK signalling. Furthermore, through the mediation of FAK phosphorylation, VEGF R2 can influence cytoskeleton reorganisation, indicating that VEGF R2 and FAK are contributors to the same signalling cascade. The findings of the present study support this and indicate that PPV does not exert its effects on this cascade. Furthermore, previous studies suggest that VEGF-activated FAK leads to increased recruitment of FAK and promotes cell migration [28,38]. However, the present study indicated that cell migration was reduced in a dose-dependent manner. This indicates that the effects of PPV on cell migration are mediated through an alternative pathway to the FAK pathway.

**Figure 9:**
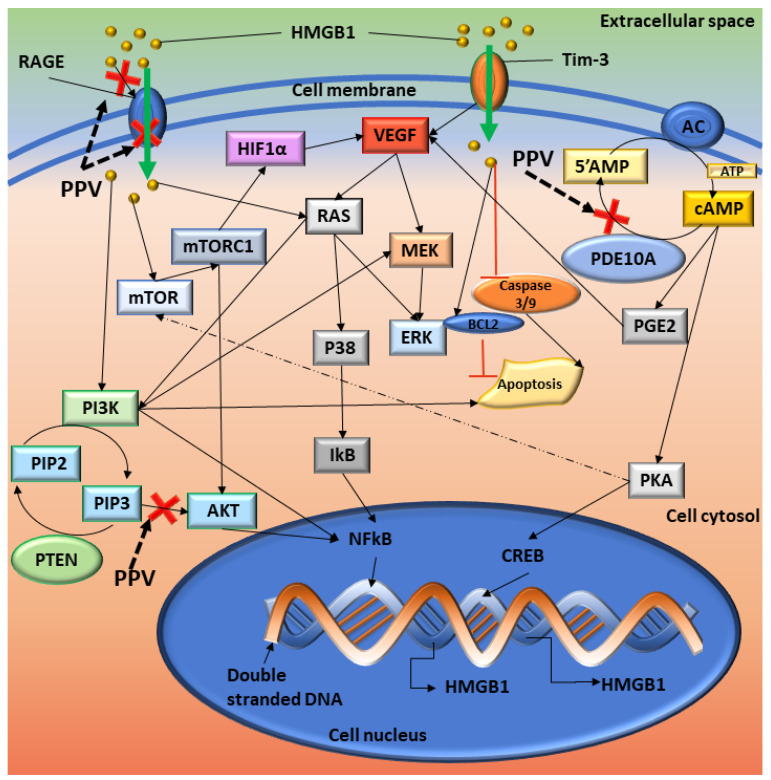
Summary of the proposed cellular signalling affected by PPV. Red crosses indicate the possible sites where PPV exerts its effects. Solid black arrows indicate the pathways and dotted arrows indicate a possible interaction. HMGB1 interacts with RAGE which induces cellular signals that affect several pathways. Once NF-kB is activated, it translocates to the nucleus where it interacts with DNA and upregulates the transcription of several genes, including HMGB1 and TNF. The interaction of HMGB1 with T Cell immunoglobulin mucin–3 (TIM-3) induces the secretion of VEGF to promote tumour angiogenesis, an immunoregulatory protein. VEGF can then stimulate MEK, which leads to ERK activation. AC controls the formation of cAMP from 5′AMP, and PDE10A degrades cAMP back into 5′AMP. Inhibition of PDE10A can lead to the build-up of cAMP, which increases the available levels of PKA and PGE2. PKA can then upregulate CREB, which increases the transcription of several downstream molecules, including HMGB1, and surviving. HMGB1 can inhibit the caspase 3/9 pathway, releasing proapoptotic inhibitors such as BAX and controls the protein levels of Bcl-2 [53,54]. Image designed by DA Gomes using Microsoft^®^ Office PowerPoint (Microsoft Office enterprise 2007, 2006 Microsoft Corporation, Redmond, WA, USA).

Tumourigenesis is a complex process with multiple different pathways including the cAMP, PI3K/Akt, FAK, and VEGF pathways (Figure 9). The PDE10A and cAMP pathways have been linked to several different survival pathways, including the PI3K/Akt and ERK pathways, which could affect VEGF and FAK signalling [18,19,20,21,22,23,24,25,26,27]. Furthermore, these pathways can influence several downstream pathways, including the ERK, mammalian target of rapamycin (mTOR), and VEGF pathways. PDE10A regulates the levels of cAMP through degradation. When PDE10A is inhibited, the accumulation of cAMP occurs. As a result, cAMP leads to the activation of protein kinase A (PKA); PKA can then activate cAMP-response element-binding protein (CREB), which leads to the upregulation of several downstream proteins, including high mobility group box 1 (HMGB1) [53,54]. Furthermore, the cAMP pathway mediates the production of prostaglandin E2 (PGE2) which induces the secretion of VEGF [22]. Studies have also shown that PKA possibly influences the levels of mTOR. However, this association needs further investigation. The inactivation of several tumour suppressor genes, including phosphatase and tensin homolog (PTEN), p53, and necrosis factor 1 (NF1), have been implicated in the regulatory associated protein of TOR (raptor)-mTOR activation, also referred to as mTOR complex 1 (mTORC1), suggesting the increase in cell growth is a result of the raptor-mTOR complex [55]. There is growing evidence of cross signalling between the cAMP pathway and the mTOR pathway [56]. Studies have suggested that cAMP can either inhibit or stimulate the formation of mTOR complexes depending on cell type [56]. Therefore, the cAMP and mTOR pathways can upregulate cell cycle progression, cell mobility, cell survival, and metastasis in several tumourous tissue types [22,23,26,56,57]. The literature also indicates that the PDE10A inhibitory effects of PPV ultimately affect the regulation of cAMP. Consequently, the antiproliferative effects of PPV may be mediated through its effects on cAMP, which may further mediate the inhibition of raptor-mTOR signalling through disruption by PKA. However, this mechanism is not fully understood [9,56]. Furthermore, upregulation of mTOR and raptor-mTOR leads to the upregulation of Hypoxia-inducible factor 1α (HIF1α), which has been shown to upregulate VEGF ligands and receptors [53,54]. It is, therefore, possible that the effects exerted by PPV observed in this study and previous research conducted by our laboratory on cell proliferation, cell migration, VEGF B and VEGF R1 is possibly mediated by the PDE10A, cAMP, mTOR, and PI3K/Akt pathways. However, further investigation into these mechanisms must be conducted [12]. 

The current study has aided in understanding the effects of non-narcotic benzylisoquinoline alkaloid PPV on tumourigenic cell lines. Data obtained in the current study will contribute to the understanding of the effects of PPV on tumourigenic cell lines in addition to their biochemical molecular targets, including VEGF receptors and ligands. Unravelling the signalling transduction induced by PPV will improve cancer researchers’ understanding of phytomedicinal compounds, contributing to the existing knowledge regarding the influence of naturally occurring compounds in tumourigenic cells.

## 4. Materials and Methods

### 4.1. Materials

#### 4.1.1. Cell Lines

The M.D. Anderson–Metastasis breast cancer-231 (MDA-MB-231) cells, alveolar adenocarcinoma (A549) cells, and human prostate adenocarcinoma (DU145) cells were obtained from the American Tissue Culture Collection (Manassas, VA, USA) [12]. All cell lines were maintained in Dulbecco’s Modified Eagle Growth medium (DMEM) supplemented with 5 mM L-glutamine, 4 mM sodium pyruvate, 3 g/L glucose, 10% heat-inactivated fetal calf serum (FCS) (56 °C, 30 min), 100 U/mL penicillin G, 100 mg/mL streptomycin and fungizone (250 mg/L) at 37 °C and 5% CO_2_ in a humified atmosphere in 75 cm^2^ tissue flasks [12].

#### 4.1.2. Chemicals and Reagents

All reagents and chemicals were purchased from Sigma Chemical Co. (St. Louis, MO, USA) and all plasticware was purchased from Lasec^®^ SA (Pty) Ltd. (Midrand, Johannesburg, Gauteng, South Africa) and supplied by Cellstar^®^, (Greiner, Germany) unless otherwise specified, as previously reported [12]. PPV was purchased from Merck (Darmstadt, Germany) and was dissolved in dimethyl sulfoxide (DMSO) to a concentration of 50 mM. Appropriate controls were used, including a negative control where cells were propagated in complete growth media as well as a vehicle-treated control where cells were exposed to growth media which contained equal volumes of the vehicle solution, DMSO, where the *v*/*v*% did not exceed 0.35% [12].

### 4.2. Methods

#### 4.2.1. Cell Migration Using Scratch Assay (Light Microscopy)

The effects of PPV on cell migration were investigated using the scratch assay. This technique involves scratching a clean line on the plastic surface of cells cultured in a monolayer. The visualisation and quantification of the scratch width after 18-, 24- and 48 h can be assessed using light microscopy (Axiovert 40 CFL microscope (Zeiss, Oberkochen, Germany)) to determine the influence of PPV on migration. The change of width of each scratch was quantified over time since migratory cells may move into the exposed surface of the scratch. If migration is inhibited, the width of the scratch will remain approximately the same [58,59].

MDA-MB-231, A549, and DU145 cells were seeded into a 24-well plate at density of 60,000 cells per well. The cells were incubated at 37 °C and 5% CO_2_ in a humified atmosphere for 24 h to allow for attachment. Each well was then scratched with a new sterile 100 µL pipette tip across the centre; a second scratch was then made perpendicular to the first scratch to form a cross in each well. Subsequently, the medium was removed, and each well was washed twice with medium to remove detached cells. Cells were then exposed to PPV (10–150 µM) since previous research showed optimal activity in cancer cell lines in this concentration range [6,7,9,11]. Negative controls for this experiment included cells propagated in complete growth medium and vehicle-treated cells. Positive controls included cells exposed to 2-ethyl-17-hydroxy-13-methyl-7,8,9,11,12,13,14,15,16,17-decahydro-6-cyclopenta[a]phenanthren-3-yl sulphamate (ESE-ol) (0.4 µM for 48 h) since previous studies indicated that ESE-ol significantly inhibits cell migration [60]. Subsequently, images were captured at 0, 18, 24, and 48 h using an Axiovert 40 CFL microscope (Zeiss, Oberkochen, Germany). The gap of each scratch was quantitatively evaluated using Image J software developed by the National Institutes of Health (Bethesda, MD, USA). 

#### 4.2.2. Detection of Vascular Endothelial Growth Factor Using ELISA (Spectrophotometry)

The effects of PPV on the expression of VEGF ligand B and receptors 1 and 2 were investigated in MDA-MB-231, A549, and DU145 cells using a VEGF B ELISA kit (Elabscience biotechnology incorporated (Houston, TX, USA)) a VEGF R1 ELISA kit and a VEGF R2 ELISA kit (Abcam plc. (Cambridge, UK). These sandwich ELISA kits involved the quantification of antigens within the sample, namely human VEGF B, VEGF R1, or VEGF R2.

MDA-MB-231, A549, and DU145 cells were seeded at a density of 500,000 cells per T25 cm^2^ flasks. The cells were incubated at 37 °C and 5% CO_2_ in a humified atmosphere for 24 h to allow for attachment. Subsequently, the cells were exposed to PPV (10–150 µM) for 48- or 72 h since previous research showed optimal antiproliferative activity in tumourigenic cell lines with these exposure conditions [6,7,9,11]. Negative controls included cells propagated in a complete growth medium and cells exposed to the vehicle solvent (DMSO). Positive controls included cells exposed to 50% PBS: 50% complete growth medium for 48 h as previous research indicates that pro-angiogenic factors such as VEGF and FAK increase in response to nutrient deprivation [33]. Cells were then trypsinised and resuspended in 1 mL of complete growth medium. Cells (1 million) were then transferred to a test tube, and samples were centrifuged for 5 min at 15,000× *g*. The supernatant was removed, and the cells were washed three times with PBS (500 µL). A freeze-thaw process involving submerging the cells in an ice bath for 10 min followed by thawing at room temperature for 10 min was repeated four times until the cells were fully lysed. Samples were centrifuged for 10 min at 1500× *g* at 4 °C. The cell fragments were then removed, and the supernatant was collected in order to carry out the assay. A standard working solution (100 µL per well) consisting of the reference standard (provided by supplier) and sample diluent (provided by the supplier) was prepared to create a concentration gradient that was added to the first two columns of the plate (0–1000 pg/mL) following the manufacturer’s instructions (Elabscience biotechnology incorporated (Houston, TX, USA) or Abcam plc. (Cambridge, UK)). The absorbance values reference standards were then used to plot a standard curve referring to the gradient curve. The lysed cell samples (100 µL) were added to the remaining wells. The plate was then covered with the sealer sheet provided in the kit before being incubated for 90 min at 37 °C. Subsequently, the liquid was removed from each well, and a biotinylated detection antibody working solution specific for VEGF ligand B, VEGF R1, or VEGF R2 (100 µL) was added to each well. The plate was then covered with the sealer sheet, and the samples in the plate were mixed gently before being incubated for 1 h at 37 °C, after which the solution from each well was aspirated. Wash buffer (350 µL) was then added to each well and left to soak for 2 min before aspirating the solution from each well and patting the plate dry against clean absorbent paper. This was repeated three times before HRP conjugate working solution (100 µL) was added to each well, and the plate was covered with the sealer sheet and incubated for 30 min at 37 °C. The solution was then aspirated from each well before wash buffer (350 µL) was added to each well and left to soak for 2 min before aspirating the solution from each well and patting the plate dry against clean absorbent paper. This washing procedure was repeated five times. Subsequently, substrate reagent (90 µL) was added to each well before covering the plate with a new sealer sheet. The plate was then incubated for 15 min at 37 °C. The plate was then wrapped in foil to protect the plate from light. Subsequently, a stop solution (50 µL) was added to each well. After which, the absorbance was measured at 450 nm using an EPOCH Microplate Reader (Biotek Instruments, Inc. (Winooski, VT, USA)). 

#### 4.2.3. Determination of FAK (Phospho) [pY397] Using ELISA (Spectrophotometry)

The literature indicates that focal adhesion tyrosine kinase (FAK) and pFAK expression is upregulated in tumourigenic cell lines. The influence of PPV on active FAK expression was therefore evaluated by means of using a FAK (Phospho) [pY397] specific ELISA kit.

MDA-MB-231, A549, and DU145 cells were seeded at a density of 500,000 cells per T25 cm^2^ flasks. The cells were incubated at 37 °C and 5% CO_2_ in a humified atmosphere for 24 h to allow for attachment. Subsequently, the cells were exposed to PPV (10–150 µM) for 48- or 72 h since previous research showed optimal antiproliferative activity in tumourigenic cell lines [6,7,9,11]. Negative controls included cells propagated in a complete growth medium and cells exposed to the vehicle solvent (DMSO). Positive controls included cells exposed to 50% PBS: 50% complete growth medium for 48 h since previous research indicates that pro-angiogenic factors such as VEGF and FAK increase in response to nutrient deprivation [33]. Cells were then trypsinised and resuspended in 1 mL of complete growth medium. Cells (1 million) were then transferred to a test tube, and samples were centrifuged for 5 min at 15,000× *g*. The supernatant was removed, and the cells were washed three times with PBS (500 µL). A freeze-thaw process that involved submerging the cells in an ice bath for 10 min followed by thawing at room temperature for 10 min was repeated four times until the cells were fully lysed. Samples were centrifuged for 10 min at 1500× *g* at 4 °C. The cell fragments were then removed, and the supernatant was collected. A standard working solution (100 µL per well) consisting of the reference standard (provided by supplier) and sample diluent (provided by the supplier) was prepared to create a concentration gradient that was then added to the first two columns of the plate (0–1000 pg/mL) in accordance with the manufacturer’s instructions (Thermofisher Scientific, (Waltham, MA, USA). The absorbance values of the reference standard were then used to plot a standard curve. The lysed cell samples (100 µL) were added to the remaining wells. The plate was then covered with the sealer sheet (provided by supplier) before being incubated for 2 h at 37 °C. Subsequently, the liquid was removed from each well and biotinylated detection antibody working solution specific for FAK (Phospho) [pY397] (100 µL) was added to each well. The plate was then covered with the sealer sheet, and the samples in the plate were mixed gently before being incubated for 1 h at 37 °C, after which the solution from each well was aspirated. Wash buffer (350 µL) (provided by the supplier) was then added to each well and left to soak for 2 min before aspirating the solution from each well and patting the plate dry against clean absorbent paper. This was repeated three times before HRP conjugate working solution (100 µL) (provided by the supplier) was added to each well and the plate covered with the sealer sheet and incubated for 30 min at 37 °C. The solution was then aspirated from each well before a wash buffer (350 µL) was added to each well and left to soak for 2 min before aspirating the solution from each well and patting the plate dry against clean absorbent paper. This washing procedure was repeated five more times. Subsequently, substrate reagent (90 µL) (provided by the supplier) was added to each well before covering the plate with a new sealer sheet. The plate was then incubated for 15 min at 37 °C. The plate was then wrapped in foil to protect the plate from light. Subsequently, a stop solution (50 µL) was added to each well. After which, the absorbance was measured at 450 nm using an EPOCH Microplate Reader (Biotek Instruments, Inc. (Winooski, VT, USA)). 

#### 4.2.4. Statistical Considerations

This study set out to determine the influence of PPV on cell migration and the expression of VEGF B, VEGF R1, VEGF R2, and FAK in a triple negative breast cancer cell (MDA-MB-231), alveolar adenocarcinoma cells (A549), and prostate adenocarcinoma cells (DU145). Three independent experimental repeats were conducted for all the techniques conducted where the mean and the standard deviation were calculated. Means were illustrated by using bar charts, and standard deviations were shown with errors bars. A *p*-value < 0.05 was calculated using the Student *t*-test for statistical significance and was indicated by an asterisk (*) using Jamovi statistical software version 1.6 (The Jamovi project (2021) (Sydney, Australia). Image J software developed by the National Institutes of Health (Bethesda, MD, USA) was used to quantify the scratch assay.

## 5. Conclusions

This study aided in understanding the influence of PPV on tumourigenic cell lines and is one of the first studies to explore the effects of PPV on cell migration, VEGF, and pFAK expression in MDA-MB-231, A549, and DU145 cells. Results indicated that cellular migration was slowed as a result of increasing concentrations of PPV, with greater effects observed in A549 cells. The effects of PPV on cellular migration, pFAK, and VEGF B, VEGF R1, and VEGF R2 were investigated to determine the influence of PPV on these downstream pathways. No significant effects were observed in expression of pFAK and VEGF R2. However, dose- and cell line-specific alterations of VEGF B and VEGF R1 expression were observed, with more prominent effects observed in A549 cells. Therefore, this study contributes to the understanding of a naturally occurring compound already in clinical use in tumourigenic cell lines and may improve the understanding and use of phytomedicinal compounds in cancer research. Understanding the mechanisms affected by PPV may aid in a better understanding of the known effects of the compound and may give rise to more effective derivatives that may be better alternatives to current compounds in use on cancer cell lines.

## Data Availability

Data is contained within the article.
